# Sarcoptic mange outbreak decimates South American wild camelid populations in San Guillermo National Park, Argentina

**DOI:** 10.1371/journal.pone.0256616

**Published:** 2022-01-21

**Authors:** Hebe del Valle Ferreyra, Jaime Rudd, Janet Foley, Ralph E. T. Vanstreels, Ana M. Martín, Emiliano Donadio, Marcela M. Uhart

**Affiliations:** 1 Administración de Parques Nacionales, Buenos Aires, Argentina; 2 Department of Medicine and Epidemiology, University of California, Davis, California, United States of America; 3 One Health Institute, School of Veterinary Medicine, University of California, Davis, California, United States of America; 4 Departmento de Patología Animal, Universidad Católica de Córdoba, Córdoba, Argentina; 5 Instituto de Investigación en Biodiversidad y Medioambiente (INIBIOMA), CONICET, Bariloche, Argentina; Universitat Autonoma de Barcelona, SPAIN

## Abstract

Sarcoptic mange epidemics can devastate wildlife populations. In 2014, mange was first detected in vicuñas (*Vicugna vicugna*) and guanacos (*Lama guanicoe*) in San Guillermo National Park (SGNP), Argentina. This study describes the temporal dynamics of the outbreak, its effects on the park’s wild camelid populations between 2017–2019, and investigates the potential source of the epidemic. From May 2017 to June 2018, transect surveys indicated a sharp decrease in the density of living vicuñas and guanacos by 68% and 77%, respectively. By April 2019 no vicuñas or guanacos were recorded on transect surveys, suggesting their near-extinction in the park. Clinical signs consistent with mange (e.g., intense scratching, hyperkeratosis, alopecia) were observed in 24% of living vicuñas (n = 478) and 33% of living guanacos (n = 12) during surveys, as well as in 94% of vicuña carcasses (n = 124) and 85% of guanaco carcasses (n = 20) examined. *Sarcoptes scabiei* was identified as the causal agent by skin scrapings, and the cutaneous lesions were characterized by histopathology (n = 15). Genetic characterization revealed that mites recovered from seven vicuñas (n = 13) and three guanacos (n = 11) shared the same genotype, which is consistent with a single source and recent origin of the epidemic. Tracing the potential source, we identified a governmental livestock incentive program which introduced llamas (*Lama glama*) in areas adjacent to SGNP in 2009, some of which had alopecic scaling consistent with sarcoptic mange. Though at the time of our study no llamas with mange were available for confirmatory sampling, we hypothesize that the introduction of mange-infected llamas may have triggered the outbreak in wild camelids. This unprecedented event in SGNP had devastating effects on dominating herbivores with potentially profound cascading effects at the community and ecosystem levels.

## Introduction

Emerging infectious diseases are caused by new pathogens or known pathogens that have recently increased their incidence or geographic distribution or have spread to new hosts [1]. In wild animals, such diseases have caused dramatic population declines, often leading to collapse and local extinction [1–5]. Sarcoptic mange, a highly contagious skin disease of mammals caused by the mite *Sarcoptes scabiei*, is an emerging disease of increasing relevance for wildlife [6].

Sarcoptic mange has been reported in at least 12 orders, 39 families and 148 species of domestic and wild mammals [6]. The initial lesions and their evolution vary between species and are linked to the immunological status of the host [7,8]. Bates [9] and Skerrat [10] describe two differentiable clinical forms of sarcoptic mange in wildlife; the hyperkeratotic form, associated with high mite loads and consistent with a type I hypersensitivity response, characterized by intense itching that leads to self-inflicted lesions by scratching and secondary infections in the skin; and the alopecic form, associated with low mite loads and characterized by loss of hair coverage consistent with a type IV hypersensitivity response. In general, the hyperkeratotic form has been reported in wild ungulates, while the alopecic form has been documented in some wild canids [7–11].

The epidemiology of sarcoptic mange can vary considerably in wild animals depending on characteristics of the geographic region, host species and population [7,8]. Mange outbreaks tend to initially have an epidemic behavior with high prevalences and mortality, later become endemic with low prevalences, and eventually disappear [7,12,13]. In some cases, mange epizootics recur in cycles of variable time, e.g., 30 years [7,12].

The origin of several wildlife outbreaks has been related to transmission between domestic and wild species [13–17]. Lavín et al. [14] demonstrated that domestic animals can be the source of infection for wildlife by inducing sarcoptic mange in Cantabrian chamois (*Rupicapra pyrenaica parva*) with mites obtained from domestic goats (*Capra aegagrus hircus*). Likewise, circumstantial evidence suggests that the mange outbreaks that decimated Iberian ibex (*Capra pyrenaica*) in Spain originated from domestic goats [15]. Recently, molecular-based studies by Moroni et al. [16], suggested the domestic origin of mange in wild ungulates in Spain, and Matsumaya et al. [17] confirmed the circulation of the same subtype of *S*. *scabiei* mite between domestic dogs (*Canis lupus familiaris*) and raccoon dogs (*Nyctereutes procyonoides*) in Japan.

Sarcoptic mange has been described in wild South American camelids, vicuña (*Vicugna vicugna*) and guanaco (*Lama guanicoe*) [18–21]. However, information on the distribution and prevalence of this disease is extremely scarce, restricted to a low number of sites, and mostly reported in gray literature. In camelids, the early manifestation of sarcoptic mange includes mild to severe pruritus with erythema, papules, and pustules, which commonly evolve to a chronic stage with crusting, alopecia and lichenification and thickening of the skin (hyperkeratosis) [22–24]. Difficulty walking and pain have been described, secondary to skin infections and fissures [25]. This may lead to a negative energy balance (particularly in seasonal climates), progressive emaciation, and limited ability to forage and evade predators [26,27].

In Argentina, the abundance and distribution of wild camelids have been impaired by hunting, competition with livestock and habitat loss, resulting in reductions of up to 40% in the original distribution of guanacos and the near-extinction of vicuñas in the mid-twentieth century [28]. Currently, guanacos and vicuñas are globally categorized as “Least Concern”, though some sub-populations are small, fragmented, and isolated, rendering them locally susceptible to stochastic events [29,30].

San Guillermo National Park (SGNP) was established to preserve the largest sympatric vicuña and guanaco populations in Argentina and represents the southern limit of the distribution of vicuñas [31]. In 2009, a governmental livestock incentive program introduced llamas (*Lama glama*) in areas adjacent to SGNP [32], some of which were diagnosed with mange. In 2014, sarcoptic mange was diagnosed for the first time in vicuñas and guanacos at SGNP [33], suggesting a link between the two events. Shortly after the detection of mange in wild camelids in the park, significant population declines were noticed, while the number of affected animals rapidly increased [33]. The abrupt nature and rapid progression of the outbreak points to a recent introduction of the mite in a naïve wildlife population [34,35]. Here we describe the mange outbreak affecting wild camelids in SGNP during 2017–19, and investigate the potential domestic origin of the mite strain involved.

## Materials and methods

### Study area

**Fig 1:**
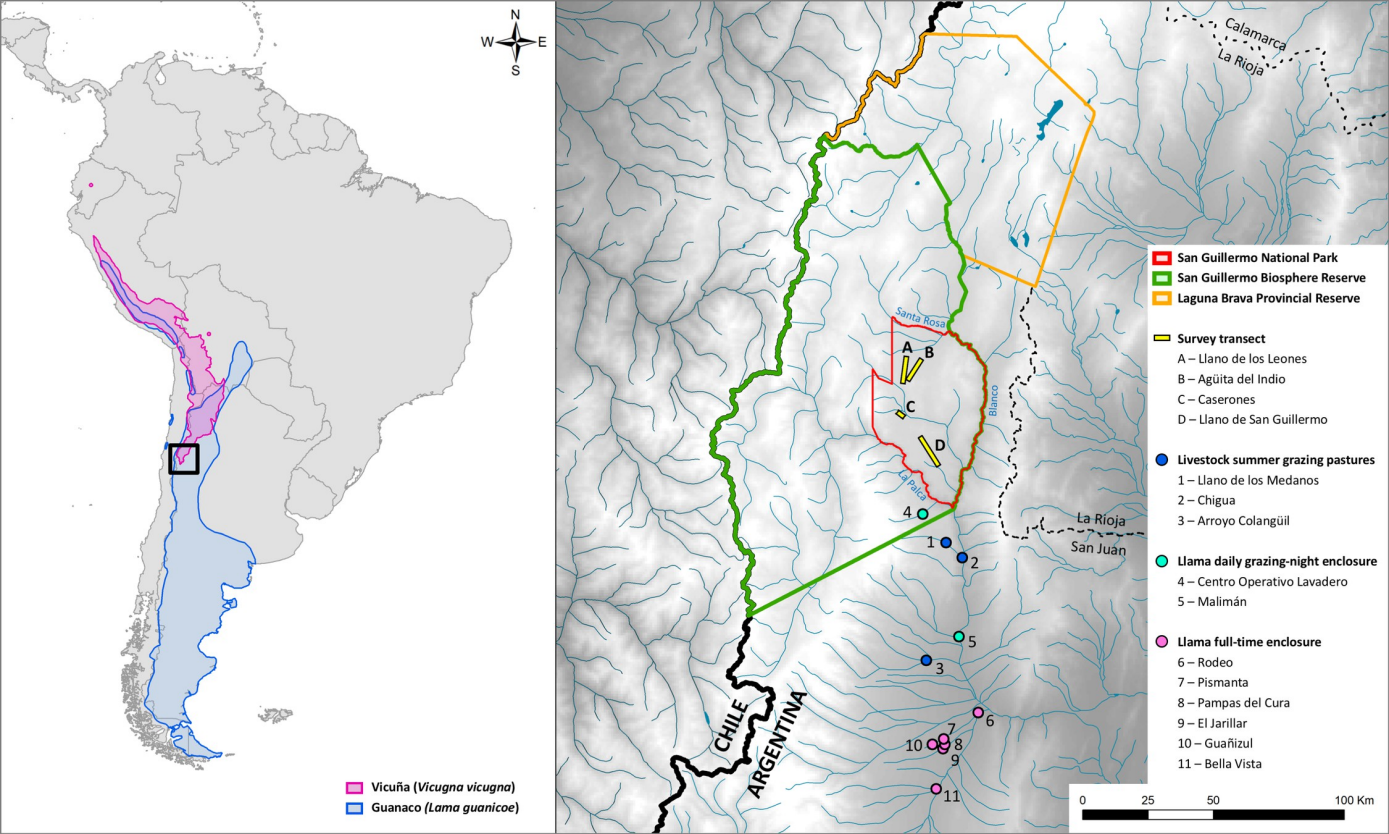
Location of San Guillermo National Park and study transects in relation to the natural distribution of vicuñas and guanacos and nearby farms and grazing areas of livestock and recently introduced llamas.

San Guillermo National Park is in the northwest corner of San Juan province in Argentina, (29°4’12"S, 69°21’0"W) (Fig 1) and covers 166,000 ha between 2,000 and 5,600 meters above sea level. San Guillermo Provincial Reserve (SGPR) (about 815,460 ha) surrounds SGNP to the northwest. Together, SGNP and SGPR make up San Guillermo Biosphere Reserve (SGBR) (Fig 1), which protects 981,460 ha of the Puna and High Andes eco-regions. SGBR practically comprises the entirety of the Iglesias Department of San Juan province [31].

Rainfall is scarce with an average 240 mm per year, concentrated in January-March. The temperature range is -23°C to 27°C; January is the warmest (average 14°C) and July the coldest month (average -1°C) [36]. SGNP is characterized by extensive open plains (81% of the study area) located at 3,400 meters above sea level, surrounded by hills and mountain peaks. These plains are traversed by narrow canyons (10–300 m wide) with steep rocky walls representing 15% of the study area, and a few isolated flooded meadows within the plains or on the riverbanks account for the remaining 4% of the study area [37].

In SGNP, the population of vicuñas is ten times bigger than that of guanacos [38]. While they both concentrate in high altitude meadows and plains, only guanacos migrate during the winter to lower altitude areas, where they might be in contact with livestock [39,40]. In 2009, some farmers nearby SGNP received llamas through a San Juan province governmental livestock incentive program (“Programa Camélidos de los Andes”) [32]. A group of these animals were temporarily housed within SGPR, at Centro Operativo Lavadero (Fig 1, site 4).

### Population density and proportion of living wild camelids infected with mange

#### Population density

After the onset of the sarcoptic mange outbreak in 2014, increasing prevalence of mange lesions and death of wild camelids were recorded in SGNP [33]. To estimate the density of remaining living vicuñas and guanacos, in our study we conducted five field surveys in May, September and December 2017, and April and June 2018. In each survey we implemented four transects following park dirt roads: Llano de los Leones, Llano San Guillermo, Caserones and Agüita del Indio (Fig 1; further details provided in S1 Table). We used binoculars (Tasco® 7 x 35 mm) and a telescope (Bushnell® 15–45 x 60 mm) to search for and observe animals, and laser rangefinders (Bushnell® Elite 1500 and Bushnell® DX 1000) to measure distances between the animals and the observer. We traveled transects at a speed of 20 km/h. When we spotted animals, we stopped and observed them for 5–10 min to count them and assess infection status (see next section). We travelled the transects twice at one day intervals and for every group of animals encountered we recorded the species, number of animals, distance from the vehicle and angle from the observer [41]. In May 2017, the transects were travelled only once due to logistical constraints (e.g., snow).

#### Mange infection

To quantify the proportion of living wild camelids with mange we conducted eight field surveys in February, May, September, and December 2017; April, June, and September 2018; and April 2019. We used the same transects but only evaluated individuals within 200 m from the transect after several trials in which we established that this was the most conservative distance at which we could confidently identify infected animals with the available telescope [42]. Notwithstanding, because incipient infections are undetectable without laboratory diagnosis which require diagnostic specimens, our visual estimates represent the minimum infected proportion [43].

**Fig 2:**
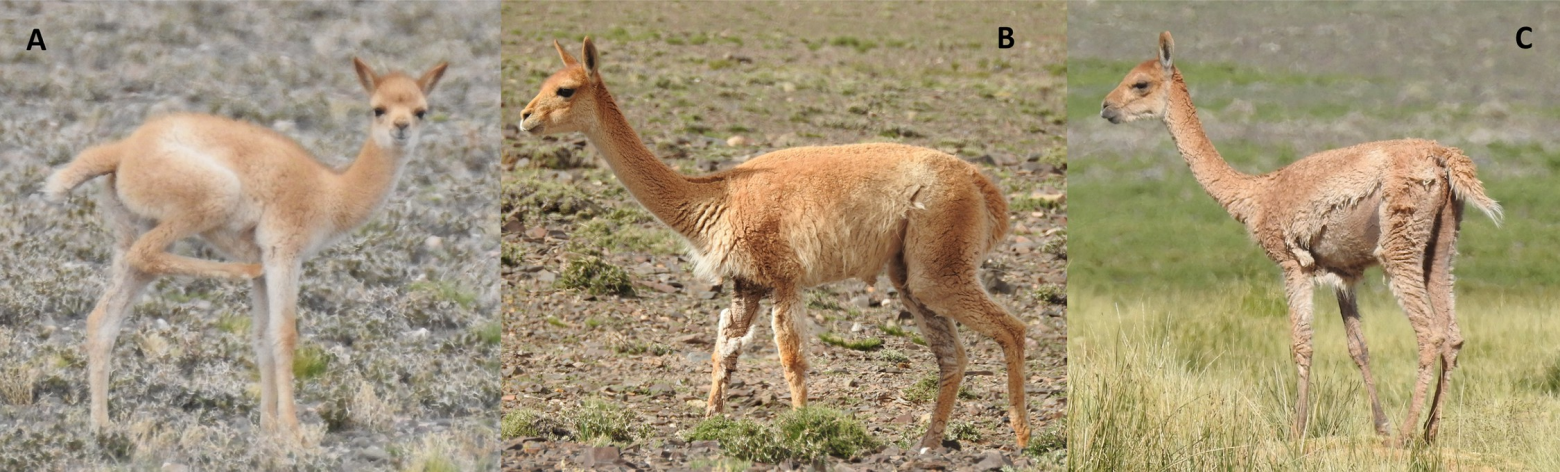
Clinical stages of mange in living vicuñas: (A) early stage, (B) advanced stage and (C) severe stage.

We identified cases of sarcoptic mange when one or more of the following signs were observed: intense scratching, difficulty walking, thickening, crusty or cracked skin, and alopecia or ruffled or detached fleece fibers [22,23,25]. According to the clinical stage of disease, we used three categories: (A) early stage, animals with obvious and severe scratching and/or individuals persistently scratching within a social group with affected animals in advanced or severe stages; (B) advanced stage, animals showing difficulty walking and/or visible injuries to the limbs; and (C) severe stage, animals with alopecia and crusting, extending to several parts of the body (Fig 2). Because the categories represent increasing severity, each level includes the signs of the previous one. To avoid inter-observer bias, all observations were made by the same person (H. Ferreyra). The age classes of living camelids were determined based on the social structure of the species and included class zero or cria, juveniles and adults [44].

#### Mange infection in wild camelid carcasses and mite collection

Wild camelid carcasses found during opportunistic searches throughout SGNP from May 2017 to June 2018 were evaluated for the presence of skin lesions consistent with mange. Carcasses were recorded only if the remains included limbs with skin and skull. Examined carcasses were identified with a cattle marker crayon, and the lower jaw was disarticulated to avoid double counts between surveys. Tooth wear and teeth replacement from the lower jaw were used to determine age class (class zero or cria, juvenile and adult) [45].

Carcasses were classified as either mange positive or negative (severity was not determined), and when possible, lesions were scraped with a sterile surgical blade. Skin scrapings were preserved in two vials, one with mineral oil for microscopy and the other with 70% ethanol for genetic characterization and multilocus genotyping [16]. *S*. *scabiei* was confirmed microscopically following standard taxonomic keys [46]. Using a magnifying glass and watchmaker’s tweezers, individual mites were recovered and placed in 70% ethanol for further characterization. Skin biopsies from mangy fresh entire carcasses were also preserved in 10% formalin for histopathology. In these cases, body condition was estimated as good, poor, or emaciated according to Van Saun [47].

### Statistical analysis

Vicuña and guanaco population densities were estimated using Distance 7.1 [48], commonly used for wild camelids in these environments [41,49]. We obtained density estimates for vicuñas and guanacos at three time periods, namely May 2017, September-December 2017, and April-June 2018. Density estimates are based on the calculation of detection probabilities which in turn depend on samples sizes >60 observations. Thus, we estimated detection probabilities for each period for vicuñas, while for guanacos we estimated detection probabilities by pooling guanaco and vicuña data due to the low number of guanacos we observed.

The proportion of mange-infected individuals and their respective 95% confidence intervals (CI) were determined for both living and dead wild camelids. Because the abundance of guanacos is low at SGNP, further statistical analysis were conducted only for vicuñas. Likelihood ratio chi-square tests were used to evaluate whether the proportion of vicuñas with mange or the proportion of disease clinical stage categories were unevenly distributed relative to age classes (excluding “not determined”), transects, and survey months. Binary logistic regression was used to evaluate whether age classes, transects and months were significant predictors of the proportion of living vicuñas with mange. Multinomial logistic regression was used to evaluate whether age classes (reference category = adult; excluding “not determined”), transects (ref = Agüita del Indio) and survey months (ref = April 2018) were significant predictors of the different stages of mange (early, advanced, severe; reference category = without mange) of living vicuñas. Odds ratios (OR) and their 95% confidence intervals were calculated for pairwise comparisons among categories of variables identified as significant (i.e. those where the OR CI interval did not include 1). Statistical analyses were conducted with Minitab 17.1.0 (Minitab Inc., State College, Pennsylvania, USA) and R 3.6.3 [50]. The datasets are available at [https://doi.org/10.5281/zenodo.5675933].

### Tracing the outbreak source

Based on the hypothesis that introduced llamas were linked to the outbreak in wild camelids, we interviewed the veterinarians who worked in the governmental livestock incentive program that brought the llamas to the study area in 2009. Interviewees were asked about the number, geographic origin, dates of arrival, and health care and husbandry of the introduced llamas over the duration of the program (two years). Additional retrospective information included where llamas were initially housed upon arrival, the names of farmers and location of properties where the llamas were delivered, and whether mange or mange-compatible lesions were noted at any time. Likewise, all llama breeders in proximity to the park were visited (Fig 1), and information was compiled on the number of llamas per herd, the characteristics of llama husbandry (intensive/semi-extensive/extensive), whether they had seen signs of sarcoptic mange, and if they were examined and treated for this disease. In the cases where llamas were still present at the farms during our study, we examined the animals for signs of mange.

To assess whether mange had been historically detected in wild camelids in the broader SGBR area, we posed the question “do you remember seeing or have you heard about mange in wildlife in this area in the last fifty years” to provincial and national park rangers, as well as to technicians from the Secretary of the Environment of San Juan province. The latter then extended the question to elderly farmers (˃60 years) in the region when they were visited for other purposes.

### Genetic characterization of mites

The Micro DNA Extraction Kit (Qiagen, Valencia, CA) procedure was used for the preparation of mite DNA from a single *Sarcoptes* mite sample according to the manufacturer’s recommendations. Prior to individual DNA extraction, dead mites were pierced with an 18-gauge needle under a dissecting microscope and digested overnight in lysis buffer and proteinase K at 56°C (Qiagen, Valencia, CA). Final DNA product from each mite was eluted in 60 μL of AE buffer. We selected 10 published microsatellite markers (SARMS 33–38, 40, 41, 44, and 45) [51] to perform multilocus genotyping of individual mites. Forward primers were labeled with HEX or 6-FAM dye (Integrated DNA Technologies, Coralville, IA) and reconstituted into 100 μM stock solutions. Primer pairs were combined into paired multiplex with 1.5–2.5 μM of each primer. We performed polymerase chain reaction (PCR) using the Qiagen 2X Type-it Multiplex PCR Master Mix, 10X multiplex primer mix (2.5 μL), DNA-free water (7 μL), and 2–3 μL DNA for a total reaction of 25 μL. Thermocycling conditions were set up as published in Rasero et al. [51]. PCR products were transferred to 96-well plates (Biotix Inc, San Diego, CA) for electrophoresis and digital measurement of length polymorphisms on an ABI 3730 analyzer (Perkin-Elmer Davis, CA) using the program STR and (Veterinary Genetics Laboratory, University of California, Davis, CA;[52]). Microsatellite scoring and allele binning were performed with the R-package MsatAllele v.1.0 [53] for R software v 3.6.1 [54].

Data was reformatted using CREATE v1.37 [55], and descriptive statistics and diversity analyses were carried out with GenAlEx v. 6.2 [56], ML-Relate [57], and R packages PopGenReport v.3.0.4 [58] and poppr v.2.8.3 [59] to determine the number of private alleles, allele frequencies, the expected (He) and observed (Ho) heterozygosity, and also to test for Hardy–Weinberg equilibrium (HWE), and partitioned components of variance using analysis of molecular variance (AMOVA). To evaluate differentiation among the *S*. *scabiei* mite populations, we calculated the pairwise F-statistic. Possible errors in multilocus genotyping due to stuttering of large allele dropout were evaluated using MicroChecker v.2.2.0.3 [60]. Null alleles were estimated using ML-Relate. P-values ≤ 0.05 were considered statistically significant.

### Ethics statement

This study was conducted in cooperation with the National Parks Administration of Argentina and personnel from San Guillermo National Park where the mange outbreak occurred, under permit number 344/2017 Administración de Parques Nacionales. No living animals were handled in this study. Specimens were only collected from animals found dead by the lead author. No animals were euthanized in this study.

## Results

### Field data

**Fig 3:**
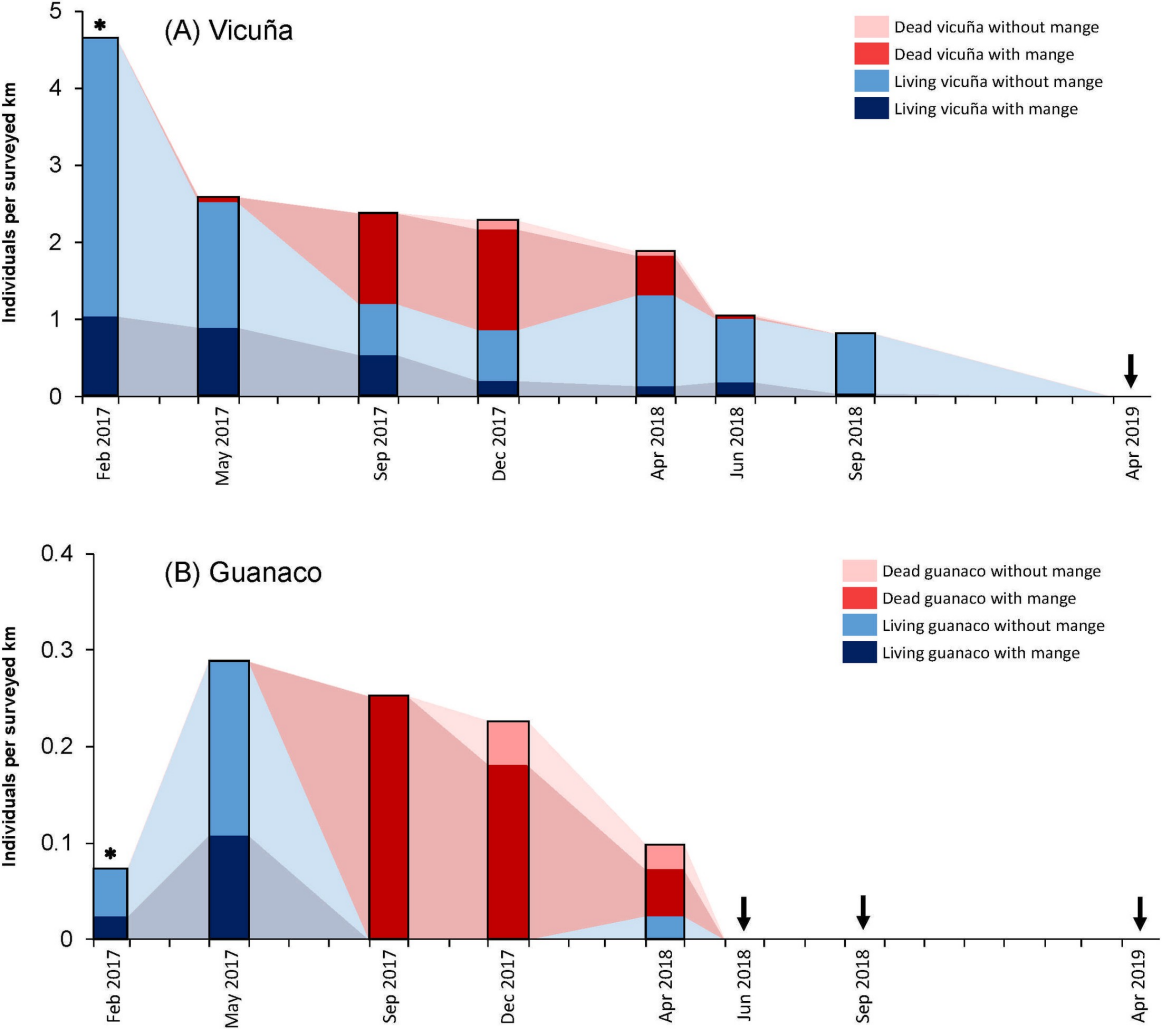
Time series of the number of living camelids observed in transect surveys and opportunistically collected carcasses with and without mange at San Guillermo National Park, February 2017 –April 2019. Asterisks indicate months when carcasses were not evaluated. Arrows represent field surveys where no individuals were recorded. Light shaded areas between bars are used to highlight the relative changes between field surveys (no data was collected in these intervals).

From May 2017 to June 2018, the population density declined from 8.89 to 2.87 individuals/km^2^ for vicuñas (68% decrease) and from 0.26 to 0.06 individuals/km^2^ for guanacos (77% decrease) at SGNP (Table 1). Fig 3 summarizes the temporal distribution of the number of wild camelids with and without mange recorded during transect surveys (living individuals) or opportunistically recorded (dead individuals). No living guanacos were seen during mange-detection transect surveys (≥ 200 m on both sides of transect) in June 2018, September 2018 and April 2019, and no living vicuñas were seen during these surveys in April 2019.

**Table 1:** Density (individuals/km^2^) of vicuñas and guanacos at San Guillermo National Park, May 2017 –June 2018.

Species	Period	Density	Standard error	Coefficient of variation (%)	95% Confidence interval	Survey effort (km)
Vicuña	May 2017	8.89	4.89	54.52	1.28–61.45	28
	Sep-Dec 2017	4.28	2.27	53	0.92–19.89	178.5
	April-June 2018	2.87	0.84	29.38	1.44–5.75	168.8
Guanaco	May 2017	0.26	1.00	97.48	0.02–3.48	28
	Sep-Dec 2017	0.23	0.14	63	0.06–13.96	178.5
	April-June 2018	0.06	0.02	38.15	0.02–0.42	168.8

**Fig 4:**
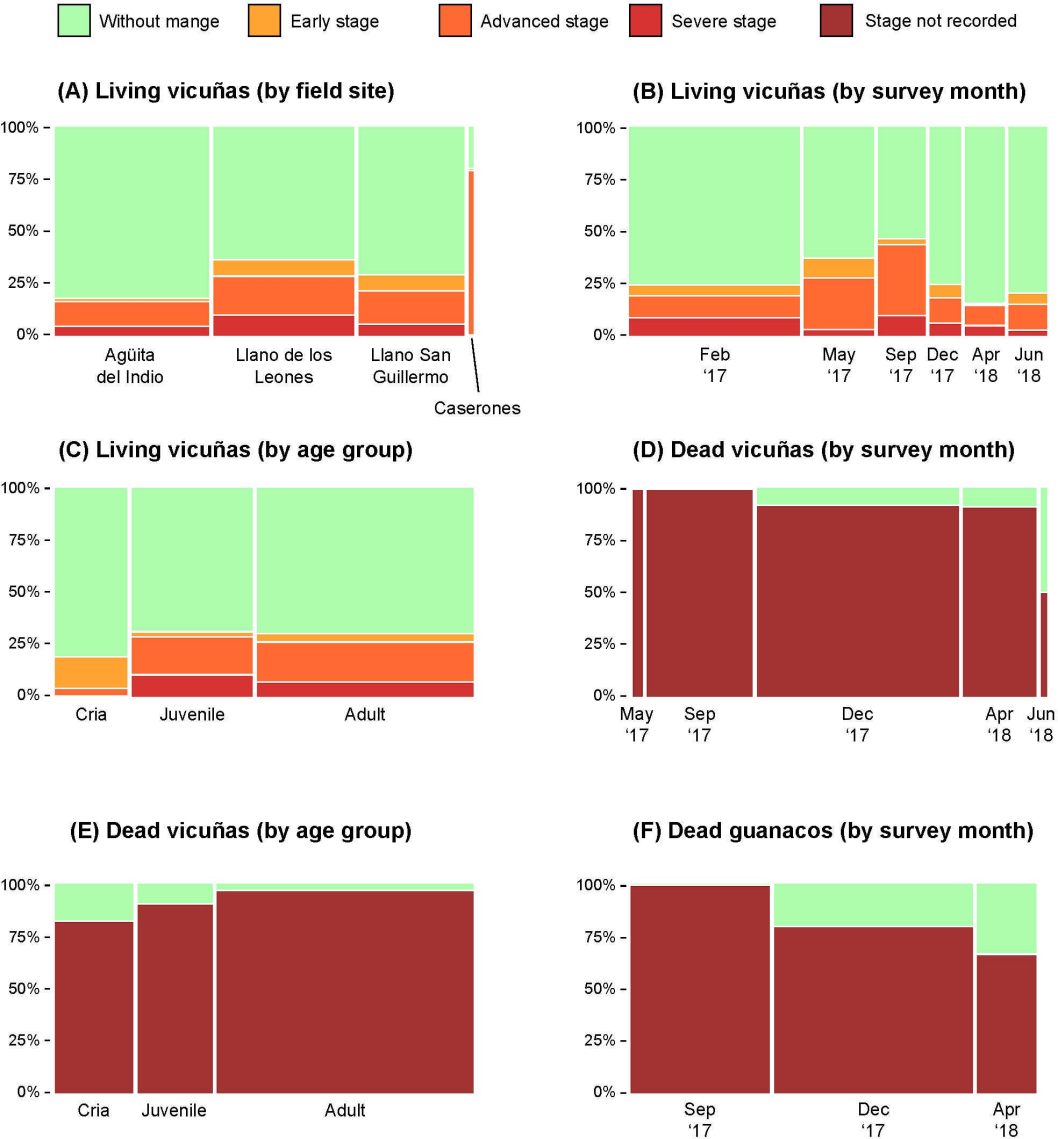
Mosaic plots of the proportion of individuals affected with mange according to the species, location, and survey month.

During the study period, 24.1% (CI = 20.3–28.2; n = 478) of living vicuñas met our case definition for mange infestation (S2 Table). Only twelve living guanacos were seen during transect surveys: three individuals in February 2017 (two adults without mange at Llano San Guillermo, one adult with mange at Caserones), eight individuals at Llano San Guillermo in May 2017 (one cria without mange, three adults with mange, four individuals of unknown age group without mange), and one individual at Caserones in April 2018 (without mange). The prevalence of mange in living guanacos (2017) was therefore 33.3% (CI = 9.9–65.1; n = 12), and all cases were in the advanced-stage category. The proportion of living and dead individuals affected with mange according to the species, transect site, and survey month are shown in Fig 4.

The proportion of living vicuñas with mange was significantly different between survey months (LRT = 31.72, df = 6, P < 0.001), transects (LRT = 16.65, df = 3, P = 0.001) and age classes (LRT = 6.747, df = 2, P = 0.034). Binary logistic regression indicated that only the survey month (P = 0.001) and transect (P < 0.001) were significant predictors of the proportion of living vicuñas with mange, whereas age class was not (P = 0.163). Specifically, the pairwise comparisons revealed that living vicuñas recorded in September 2017 were more likely to have mange than those recorded in February 2017 (OR = 4.02), April 2018 (OR = 11.26) and September 2018 (OR = 9.09) (95% CIs provided in S3 Table). Additionally, living vicuñas recorded at Caserones (OR = 5.77), Llano de los Leones (OR = 4.59), and Llano San Guillermo (OR = 3.05) were more likely to have mange than those recorded at Agüita del Indio (95% CIs provided in S3 Table).

Among living vicuñas with mange, the proportion of individuals in each disease stage category varied significantly relative to the survey months (LRT = 50.77, df = 18, P < 0.001), transects (LRT = 22.81, df = 6, P < 0.001; “Caserones” omitted) and age classes (LRT = 40.45, df = 6, P < 0.001) (S4 Table). Multinomial logistic regression for living vicuñas revealed that: (a) crias were more likely to present early stage disease (OR = 4.86) and less likely to present advanced stage disease (OR = 0.14) relative to adults; (b) individuals recorded in February 2017 were more likely to present advanced stage disease than those recorded in May 2017 (OR = 3.16), September 2017 (OR = 11.02) and June 2018 (OR = 7.85); and (c) individuals recorded at Agüita del Indio were less likely to present early stage disease than those recorded at Llano de los Leones (OR = 0.08) and Llano San Guillermo (OR = 0.06), less likely to present advanced stage disease than those recorded at Caserones (OR = 0.08) and Llano de los Leones (OR = 0.22) and less likely to present severe stage disease than those recorded at Llano de los Leones (OR = 0.18) (95% CIs provided in S4 Table).

For opportunistically-examined carcasses, 93.5% of vicuñas (CI = 87.7–97.2; n = 124) and 85.0% of guanacos (CI = 62.1–96.8, n = 20) met our case definition for mange infestation (Table 2). The overall proportion of dead individuals with mange was similar in vicuñas and guanacos (LRT = 1.485, df = 1, P = 0.223). The proportion of dead vicuñas with mange was similar among survey months (LRT = 4.725, df = 2, P = 0.094; “May 2017” and “June 2018” were omitted from this analysis due to insufficient sample size) and age classes (LRT = 5.682, df = 2, P = 0.058) (Table 2). During carcass searches, most specimens found were skeletal remains. Only five animals (four vicuñas and one guanaco), were found whole and fresh (recently predated by cougar *Puma concolor*). These five carcasses were classified as in an advanced clinical stage of mange and were all in good body condition.

**Table 2:** Number and proportion of mange in examined vicuña and guanaco carcasses at San Guillermo National Park, May 2017 –June 2018.

Category	Vicuña			Guanaco		
	Individuals examined	Individuals with mange	Proportion (95% CI)	Individuals examined	Individuals with mange	Proportion (95% CI)
*Age class*						
Cria	23	19	82.6% (61.2–95.1)	1	1	100% (2.5–100)
Juvenile	22	20	90.9% (70.8–98.9)	2	2	100% (15.8–100)
Adult	75	73	97.3% (90.7–99.7)	15	13	86.7% (59.5–98.3)
Not determined	4	4	100% (39.8–100)	2	1	50% (1.3–98.7)
*Month*						
May 2017	3	3	100% (29.2–100)	0		
September 2017	33	33	100% (89.4–100)	7	7	100% (59–100)
December 2017	63	58	92.1% (82.4–97.4)	10	8	80% (44.4–97.5)
April 2018	23	21	91.3% (72–98.9)	3	2	66.7% (9.4–99.2)
June 2018	2	1	50% (1.3–98.7)	0		
**Total**	124	116	93.5% (87.7–97.2)	20	17	85% (62.1–96.8)

### Diagnostic analysis

**Fig 5:**
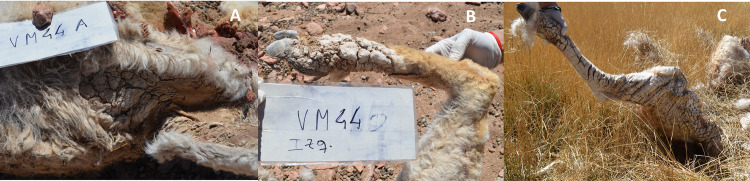
(A) vicuña carcass with scabs and deep cracks in the axillary area; (B) vicuña carcass with scabs and deep cracks on hind limb; (C) guanaco carcass with scabs and deep cracks along the hind limb and groin.

Macroscopically, the lesions in five whole carcasses examined were characterized as the hyperkeratotic or crusty form of the disease, with lichenification and thickening of the skin, presence of whitish crusts with deep cracks, and alopecia. The lesions were mostly located in body areas with less fiber coverage, namely groin, axilla, lower abdomen, medial limbs, perineum, and face (Fig 5). A total of 24 skin scrapings preserved in mineral oil, recovered from 20 vicuñas and four guanacos, showed the presence of abundant mites identified as *S*. *scabiei* [46].

Histology from 14 vicuña and one guanaco carcasses revealed typical sarcoptic mange lesions with abundant mites in all specimens. Histological findings were consistent with chronicity such as hyperplasia of the epidermis and of sebaceous glands (15/15), collagen sclerosis (12/15), as well as acute changes like presence of inflammatory cells (neutrophils, eosinophils) and congested blood vessels in all cases (15/15, 100%) (S1 Fig). Lesions identified as chronic histologically were more common in the axillary and inguinal regions of the body and coincided with areas where the greatest thickening of skin with crusts were observed macroscopically.

### Tracing the outbreak source

Four of five veterinarians interviewed participated in the governmental livestock incentive program (“Camélidos de los Andes”) between 2009 and 2011. The fifth veterinarian runs a large-animal practice in the town of Rodeo, near SGNP (see Fig 1). According to their records, 156 llamas entered San Juan province between 2009–2011 from Jujuy and Catamarca provinces (900 and 300 km north of San Juan, respectively). Veterinarians reported that llamas were initially confined to a community pen in Rodeo where mange was detected in at least two animals upon arrival from Jujuy province in 2009 (S2 Fig). Infected llamas were reportedly treated with ivermectin. Llamas were then given to local farmers (all within Iglesias department) between 2009–2011. About 15 llamas were not claimed by farmers and were placed under temporary care of provincial park rangers at a ranger post, Centro Operativo Lavadero (Lavadero), within SGPR.

During our study period, seven private farms in Iglesias department that housed llamas since 2009 were identified (Fig 1). The farmer in Malimán (since 2009) and the rangers in Lavadero (mentioned above, only temporarily) allowed llamas to graze freely. Llamas in Lavadero were reportedly moving about 8 km to the northwest into SGNP on a daily basis, and in Malimán, their space use overlapped with that of guanacos. According to two interviewees, no mange cases were observed in llamas at these two sites, although there was no sustained veterinary care of the herds due to the expiration of the government program by 2013–2014. At the time of our study, we visited all farmers currently rearing llamas (n = 7) yet failed to identify mange-infested llamas for mite recovery. This prevented us from genetically comparing llama mites from those recovered from wild camelids in the park (see section below).

Finally, the extended interviews revealed that in the last 20 years there were no outbreaks of mange in non-camelid livestock in proximity of SGNP or SGBR. Likewise, there were no previous reports of sarcoptic mange outbreaks in wild camelids in the area at least in the last 50 years since SGPR was established.

### Mite characterization

No mange-infested llamas were identified during our study. A total of 24 mites were selected for molecular identification from the skin scrapings of dead vicuñas and guanacos; 13 mites collected from seven vicuñas and 11 mites collected from three guanacos. Sixteen alleles were detected across 10 microsatellite loci. Depending on the loci, allele count ranged from one (SARM-36 and 38) to three (SARM-33 and 40). A total of 6 private alleles (i.e. alleles found only in one population and among the broader collective populations of study) were detected and distributed among eight loci (SARM-33, 35, 37, and 40). The distribution and allele frequencies among populations of *S*. *scabiei* mites according to the host is presented in Table 3.

**Table 3:** Frequency of alleles by population.

Locus	Allele	Vicuña Mites	Guanaco Mites
**SARM-33**	**N**	**12**	**11**
	**245**	0.083^†^	0.000
	**247**	0.875	1.000
	**274**	0.042^†^	0.000
**SARM-45**	**N**	**13**	**11**
	**194**	1.000	1.000
**SARM-35**	**N**	**12**	**11**
	**136**	1.000	0.818
	**138**	0.000	0.182^†^
**SARM-38**	**N**	**13**	**11**
	**211**	1.000	1.000
**SARM-34**	**N**	**13**	**11**
	**209**	1.000	1.000
**SARM-44**	**N**	**13**	**11**
	**270**	1.000	0.909
	**272**	0.000	0.091^†^
**SARM-40**	**N**	**13**	**11**
	**248**	0.154^†^	0.000
	**250**	0.846	1.000
**SARM-41**	**N**	**13**	**11**
	**236**	1.000	1.000
**SARM-36**	**N**	**13**	**11**
	**272**	1.000	1.000
**SARM-37**	**N**	**13**	**11**
	**180**	0.923	1.000
	**274**	0.077^†^	0.000

Distributions of allele frequencies in 10 microsatellite loci among *Sarcoptes scabiei* mite populations by host, vicuña and guanaco (allele sizes are in base pairs). N is the number of mites collected and genotyped from seven vicuñas and three guanacos at each allele. Private alleles are denoted with “^†^”.

Vicuña-derived mites had more total alleles detected overall (n = 14) compared to mites collected from guanacos (n = 12); however, both mite camelid-derived populations displayed relatively low allelic richness (R_vicuña_ = 1.35, R_guanaco_ = 1.19, Table 4). Further, mites from vicuñas and guanacos presented relatively few alleles with a low occurrence of polymorphisms, 30% polymorphic loci in vicuña-derived mites and 20% in guanaco-derived mites. Fixed alleles were detected for both vicuña and guanaco-derived mites at SARM-34, 36, 38, 41, and 45 (S5 Table). Fixed alleles were also observed for vicuña-derived mites at SARM-35, 37, and 44, while additional fixed alleles for guanaco-derived mites were detected at SARM-33 and 40. Values of expected (He) and observed (H_o_) heterozygosity were also low for mites collected from vicuñas (H_e_ = 0.063, H_o_ = 0.024) and guanacos (H_e_ = 0.046, H_o_ = 0.055).

**Table 4:** Characteristics of genetic variability of *Sarcoptes scabiei* obtained from vicuña and guanaco carcasses in San Guillermo National Park.

Mite host	No. of mites	R	No. of polymorphic loci	H_o_	H_e_
Vicuña (*n* = 7)	13	1.35	3	0.024	0.063
Guanaco (*n* = 3)	11	1.19	2	0.055	0.046

*Abbreviations*: *n*, number of individuals sampled; R, allelic richness; H_o_, observed heterozygosity; H_e_, expected heterozygosity.

Mites from guanacos showed no significant deviations from the Hardy-Weinberg equilibrium (HWE), while mites from vicuñas had significant HWE departures at SARM-33 (P = 0.032) and SARM-40 (P = 0.004). AMOVA analysis showed the highest percentage of variance to occur within samples (57.7%, P = 0.04) rather than between populations (6.35%, P = 0.01). Pairwise F_ST_ values demonstrated both populations were closely related (F_ST_ = 0.054, P = 0.025).

## Discussion

Sarcoptic mange is an emerging global wildlife disease. Recent reported cases worldwide reflect broad geographic spread, an increase in host species and greater virulence, and have been associated with population declines [6]. Here we report an outbreak of mange with a devastating effect on wild camelid populations within a protected area and its potential link with introduction of domestic llamas in the vicinity.

This study, which spanned a period of 26 months (February 2017 –April 2019), took place at an advanced stage of the epidemic, when the population reduction was most drastic. By the end of this study, vicuñas and guanacos had nearly disappeared from the park (Tables 1 and S2, Fig 3). Explosive outbreaks such as this are rare for mange, yet a similar event was reported in the Iberian ibex of the Cazorla, Segura and Las Villas Natural Park, in Spain [15]. In the ibex, a quick rise to 81% prevalence of sarcoptic mange was paired with a population reduction of 95%. Other examples of significant population-level impacts include an outbreak in non-native wild Barbary sheep (*Ammotragus lervia*) also in Spain, causing an 86% population decrease [13] and mortality rates of over 80% leading to demographic collapse in chamois (*Rupicapra rupicapra*) in Italy [61]. There is little data available on mange in wild South American camelids, but reports mostly suggest endemic status with low prevalences associated with domestic camelid cohabitation and/or live-shearing practices [19–21,23]. Notwithstanding, an increasing trend in mange distribution and infection prevalence has been recently reported for vicuñas in several countries across their range [30,62,63].

Despite the continuous numerical decline in wild camelids in the park, mange persisted at the end of this study, albeit at low rates. This suggests that mechanisms independent of density were involved in transmission, such as frequency-dependent mechanisms (e.g., mating behavior), that allow a pathogen to continue to spread even when population size declines to the point of near local extinction [64,65]. Indirect transmission through contact with fomites [64,66,67] is also possible. In particular, the role of communal sites such as dust baths or other elements of the environment like shrubs (in this study it was observed that animals used hard vegetation to scratch) in the transmission of mange remain unknown. The severe hyperkeratotic or crusted clinical form of mange observed in this outbreak is characterized by high load of mites and is thus highly contagious [68]. The survival of the mite outside the host with the ability to infect is a key factor for transmission, yet it has not been thoroughly investigated under field conditions. Arlian et al. [69] demonstrated that, generally, higher relative humidity values and lower temperatures favored survival, and Loredo et al. [70] showed longer than expected mite survival in winter when temperatures are cooler and humidity is higher. Ibrahim and Abu-Samra [71] experimentally observed more severe lesions in animals infected with *S*. *scabiei* when incorporating moisture into the skin, which was probably due to weakening of the stratum corneum of the skin and a greater survival of the mite. Retrospective, long-term evaluations of climate conditions prior to and during mange outbreaks in wildlife would allow to detect associations with outbreak severity.

While seasonal trends in mange outbreaks have been described in European ungulates [72–74], the reasons are not clear. It has been suggested that mites may be more active in the spring due to seasonal variations in their fertility [75]. This could be coupled with poor physical condition of hosts at the end of winter and thus greater vulnerability, as well as greater interaction and probability of contagion during the reproductive season [15]. In the case of vicuñas at SGNP, there was no clear seasonal trend since a higher proportion of mange was only seen in spring 2017, but not in 2018. However, our study period was too short to confirm or discard seasonality in the epidemiology of the disease during this outbreak. In Chile, a higher prevalence of mange in wild vicuñas has been reported for spring-summer [76]; however, there are no long-term studies that confirm seasonality in other locations in South America.

Mangy wild camelids were seen throughout the study period at SGNP. In living animals, distant observation up to 200m allowed us to detect cases and categorize the stages of disease, coinciding with the experience of Leon Vizcaíno et al. [15] and Arenas et al. [42] for large ungulates. In the case of carcasses, we were able to diagnose a high proportion of mange-infestations despite examination of mostly limbs with skin remains, which may have missed infection in other parts of the body. The occurrence of mange in living vicuñas was similar across age classes, but severity varied, and severe stages were not observed in crias. Because crias were seen nursing from severely ill mothers, it is possible that lack of maternal care led to mortality of this age class before mange progressed. A higher proportion of vicuñas in advanced stage of the disease, at which there is visible difficulty in their movements, was seen in the meadow of Llano de los Leones. Meadows are also the preferred hunting sites for puma [77,78], which may explain the steady numerical declines and the removal of animals before they reached severe stages of disease. Preliminary data show that the percentage of puma-killed mangy camelids (n = 392) increased from 5 to 90% in 24 months at the outbreak onset [33].

Spatially, the outbreak was initially detected in both SGNP and the larger SGPR. However, over time, infected camelids were observed in adjacent, outside park boundary areas. For example, mange-infected vicuñas and guanacos were reported to the north of the park in 2016 (La Brava Reserve in La Rioja province, Fig 1) and infected guanacos were seen to the northeast, in San Juan province in 2018. While vicuñas are naturally restricted to high altitude locations, guanacos in this region are migratory and more prone to overlap with livestock, and recently, with introduced llamas. In addition to the altitudinal migration of guanaco [79,80], they have been reported to have wide home-range areas (1853 km^2^) [40]. This makes guanaco more prone to contact with livestock and confers them a biological potential to disperse mange if infected [81]. Guanacos could have thus acted as a bridge species for the transmission of the mite during their migration towards the high Andes inhabited by vicuñas.

Interviewed veterinarians reported that the only cases of mange near SGNP in the last 20 years occurred in imported llamas in 2009, when they were first brought to San Juan province. Mange was specifically reported in a llama herd from Cieneguillas, Jujuy province, a site where mange is common in domestic and wild camelids [20]. The veterinarians reported that one of the infected llamas was treated for mange and then handed to a farmer in Malimán, who allowed his herd to graze freely and comingle with free-ranging guanacos. This situation may have also occurred in the higher-altitude Lavadero area, the ranger post adjacent to SGNP, where both wild vicuñas and guanacos are present. While interviewee responses allowed us to locate all the llama delivery sites which still conserved herds at the time of our study, this information was incomplete and many years old; thus, we cannot rule out the existence of more sites of spatial overlap between wild and domestic camelids in the past. From the extended interviews in San Juan, it is evident that at least in the last half century, mange had never been reported in wild camelids in the SGBR or its area of influence, confirming this is a new, emerging event.

The guanaco and vicuña mites evaluated in this study presented highly homologous genotypes, being mostly monomorphic in all loci and most of them sharing the same alleles with very little genetic variability. The observed (Ho) and expected (He) heterozygosity in guanaco and vicuña mites remained within expected parameters, suggesting that they were in HWE [82]. In HWE populations, allele and genotype frequencies are assumed to remain constant from generation to generation in the absence of other evolutionary influences (migration, mutation, selection, gene drift) [82], suggesting that the mange epidemic described here originated from a single source and a single introduction event. Low genetic diversity is common in newly introduced pathogens [34] and consistent with the rapid spread of an emerging pathogen [35]. Unfortunately, at the time of our study there were no llamas with mange in the area, which precluded us from further exploring this species as a source of mite introduction. Future studies should apply molecular techniques such as single nucleotide polymorphisms to clarify the phylogenetic relationships, host preference of mites, mechanisms of propagation, and the source and origin of infestations [6,83]. Recently, molecular epidemiological studies have informed on domestic animal sources in wildlife mange outbreaks [84] as well as on transmission between domestic and wild animals [17].

Disease control interventions in wildlife are challenging, costly and often unsustainable in the long term [85]. High conservation value species affected by mange have been treated with medication (e.g., ivermectin, fluralaner) [86–89]. Yet data on efficacy, pharmacokinetics, and other basic aspects of these therapeutic drugs is lacking for most wild species, and few interventions have succeeded in eliminating mange after a single dose or been possible in free-ranging conditions [90,91]. In wild settings, relay toxicity of drug residues to predators and scavengers must be considered, as well as environmental toxicity. The latter is rarely acknowledged, despite recognized impacts on key biotic elements such as dung beetles (e.g., ivermectin and metabolites) [92,93]. In the case reported here, interventions other than treatment (e.g., removal of infected animals) may have been possible at the onset, but were apparently not considered necessary, possible, or relevant (e.g., the event was considered “natural” population regulation). That mange is a common disease, caused by a relatively low-pathogenicity agent rarely causing population level consequences may have been misleading for managers, until interventions were no longer feasible.

Introduction of mange by domestic animals has been suspected in several wild ungulate outbreaks in Spain including in Barbary sheep, Iberian ibex and Cantabrian chamois [13,15,94]. Health risks associated with movements of livestock near national parks are rarely considered in Argentina, and there is little communication between the conservation and agriculture sectors. Thus, livestock incentive programs like the one described here occur under a totally separate set of priorities, agencies, and legislation, with no overlap or consultation with the environmental sector. Moreover, sarcoptic mange is not a mandatory reportable disease in Argentina, so records on species and areas affected are not available. Although we were unable to confirm a domestic animal source in the outbreak reported here, the most efficient management approach going forward would be to avoid the presence of livestock within protected areas. Likewise, adequate disease surveillance, prevention and control practices in conservation units that allow livestock grazing should be implemented.

The establishment of a llama breeding program, which included their introduction to the SGPR without previous consideration of the disease risks due to their taxonomic proximity with the native camelids protected there, plus the discontinuation of veterinary care for introduced animals, carried a high cost for vicuñas and guanacos in SGNP. Despite this being a protected area, since the outbreak the local extinction of wild camelids in SGNP is a real possibility. Disease outbreaks can have strong numerical effects on dominant herbivores with effects at the community and ecosystem levels [95]. In our study site, the catastrophic decline of the vicuña population is predicted to benefit grasslands in habitats where vicuñas exert high grazing pressure [96], negatively affect puma and avian scavenger populations which depend heavily upon vicuñas and vicuña carcasses as a food resource [97–99], and alter nutrient transport and dynamics [100]. Only science-based, comprehensive, and multi-sectorial policies that bridge the environment and livestock sectors can herald a better future for the health of all species.

## Conclusions

Sarcoptic mange had an epidemic behavior with a devastating effect on wild camelids at SGNP. At the end of this study, a scenario of high risk for local extinction of vicuñas and guanacos in this protected area was evident. Several factors may have contributed to the rapid spread of mange in SGNP, including a high sensitivity of the animals to the mite evidenced by a severe clinical form of the disease; the social nature and gregarious behavior of camelids; and the initial high densities of camelids in the park, which would have favored contact between individuals and significant spatio-temporal overlap between healthy and sick animals. Mange infection and the related high susceptibility to puma predation were determining factors in the population collapse observed.

A series of considerations support the hypothesis of the origin of the outbreak in introduced llamas: a) from the interviews, two sites of spatio-temporal overlap between introduced llamas and wild camelids were detected within and around SGBR; b) there were temporal coincidences between the launch of llama production in San Juan (2009–2014) and the detection of the first cases of mange in native camelids in the park (2014); c) sarcoptic mange is a frequent problem in llamas in at least one of the sites of origin of the introduced animals (Cieneguillas); d) mange was diagnosed in some llamas entering San Juan from Cieneguillas, and it is possible that further unnoticed cases occurred, either due to lack of reports or subclinical and/or mild infestations; e) interviews suggest that mange has not been a problem in livestock in the last two decades in SGNP’s area of influence, and no outbreaks of mange have been reported in native camelids in the area in the last five decades; f) the genetic characteristics of the mites recovered from guanacos and vicuñas suggest that it was a recent introduction, with no time to co-evolve with SGNP wild camelids, supporting that the mite is exogenous to the affected population; g) the aggressive and epidemic behavior in SGNP vicuñas and guanacos suggests no prior contact with the disease (“naïve” population).

The transmission of diseases between wild and domestic animals will be an increasing challenge at the interface. In Argentina, sarcoptic mange is not notifiable in livestock, but should be considered by the national veterinary service so that efficient disease control mechanisms are implemented in interprovincial animal movements, particularly when they involve protected areas. Proper sanitary management of domestic animals will always be a more reasonable and feasible strategy than trying to contain epidemics in wild populations. With the loss of the largest and main herbivores in the SGNP system, large ecosystem-wide changes are expected in the park. Long-term monitoring will provide valuable information to assess the resilience of the system in response to disease-driven extinction of key species.

## Supporting information

S1 Fig Histology from skin recovered from vicuñas: 1) Intraepidermal *Sarcoptes scabiei* mite, 2) marked epidermal hyperkeratosis, 3)Sebaceous gland hyperplasia, 4) lymphoplasmacytic infiltrate. (Photos: A.M. Martin). (TIF)

S2 Fig A and B: Llamas with alopecic scaling and crusts on forelimbs indicative of sarcoptic mange (Photo: M. Ciallela). These photographs were taken upon arrival of llama to Rodeo (San Juan province) from Cieneguillas (Jujuy province) in 2009. C: mange-free llama feet (Photo: H. Ferreyra). (TIF)

S1 Table Mange detection survey effort (km) per transect in each month. The Agüita del Indio transect was not surveyed on May 2017 due to road blockage by excessive snow.
(DOCX)

S2 Table Number and proportion of living vicuñas with mange recorded during transect surveys at San Guillermo National Park, February 2017 –April 2019. The stages of clinical disease were categorized as: (A) early stage, scratching evident and/or individuals persistently scratching within a social group with affected animals in advanced or severe stages; (B) advanced stage, difficulty walking and/or visible injuries to the limbs; and (C) severe stage, alopecia extending to several parts of the body. Because the categories represent increasing severity, each level includes the signs of the previous one.
(DOCX)

S3 Table Odds ratio of different variable categories with regards to the occurrence of mange in living vicuñas. Interpretation example: “living vicuñas observed in December 2017 were 5.33 times more likely to present mange than living vicuñas observed in April 2018.” Asterisks indicate significant differences among levels (P < 0.05).
(DOCX)

S4 Table Odds ratio of different variable categories with regards to the occurrence of different clinical stages of mange in living vicuñas. Interpretation example: “living vicuñas of the “cria” age group were 4.68 times more likely to present early stage mange than living vicuñas of the “adult” age group.” Asterisks indicate significant differences among levels (P < 0.05).
(DOCX)

S5 Table Chi-square test summary comparing observed and expected heterozygosis for the Hardy-Weinberg equilibrium in mites from guanacos and vicuñas. (DOCX)
