# Supplementary figures and images for: Sarcoptic mange outbreak decimates South American wild camelid populations in San Guillermo National Park, Argentina
Source: PLoS One. 2022 Jan 21;17(1):e0256616. doi: 10.1371/journal.pone.0256616 (PMC8782313; doi:10.1371/journal.pone.0256616)

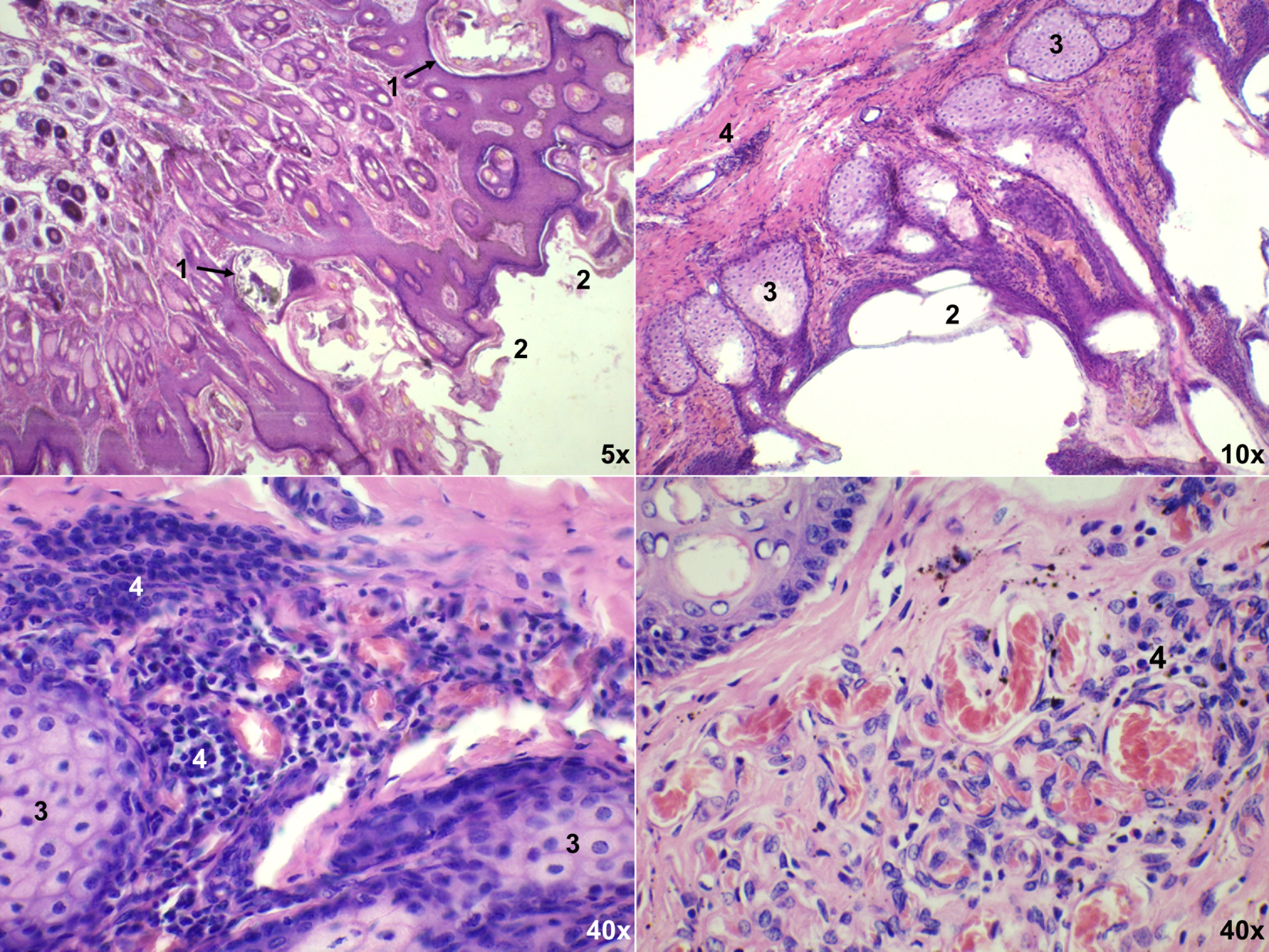

Supplement: S1 Fig — (TIF) [file pone.0256616.s001.tif]

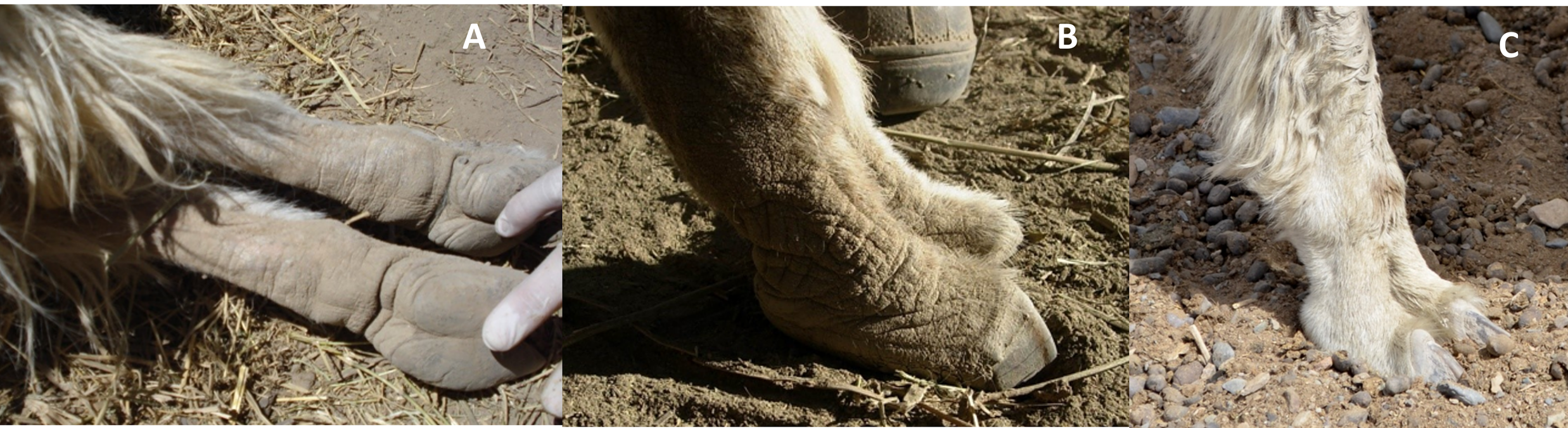

Supplement: S2 Fig — (TIF) [file pone.0256616.s002.tif]
