# Supplementary material for: Sarcoptic mange outbreak decimates South American wild camelid populations in San Guillermo National Park, Argentina
Source: PLoS One. 2022 Jan 21;17(1):e0256616. doi: 10.1371/journal.pone.0256616 (PMC8782313; doi:10.1371/journal.pone.0256616)
Supplement: S1 Table — The Agüita del Indio transect was not surveyed on May 2017 due to road blockage by excessive snow. (DOCX) [file pone.0256616.s003.docx]

**Table S1: Mange detection survey effort (km) per transect in each month. The Agüita del Indio transect was not surveyed on May 2017 due to road blockage by excessive snow.**

| **Survey** | **Llano de los Leones** | **Agüita del Indio** | **Caserones** | **Llano San Guillermo** |
| --- | --- | --- | --- | --- |
| Feb 2017 | 9.6 | 13 | 5 | 13 |
| May 2017 | 10.1 | ─ | 4.7 | 13 |
| Sep 2017 | 12 | 13 | 5.3 | 14 |
| Dec 2017 | 9.9 | 12 | 5.4 | 13.3 |
| Apr 2018 | 10 | 15 | 4 | 12 |
| Jun 2018 | 10.1 | 14.9 | 5 | 12.5 |
| Sep 2018 | 9.4 | 14.5 | 4.8 | 13.3 |
| Apr 2019 | 10.1 | 13 | 5 | 13 |
