# Supplementary material for: Sarcoptic mange outbreak decimates South American wild camelid populations in San Guillermo National Park, Argentina
Source: PLoS One. 2022 Jan 21;17(1):e0256616. doi: 10.1371/journal.pone.0256616 (PMC8782313; doi:10.1371/journal.pone.0256616)
Supplement: S2 Table — The stages of clinical disease were categorized as: (A) early stage, scratching evident and/or individuals persistently scratching within a social group with affected animals in advanced or severe stages; (B) advanced stage, difficulty walking and/or visible injuries to the limbs; and (C) severe stage, alopecia extending to several parts of the body. Because the categories represent increasing severity, each level includes the signs of the previous one. (DOCX) [file pone.0256616.s004.docx]

**Table S2: Number and proportion of living vicuñas with mange recorded during transect surveys at San Guillermo National Park, February 2017 – April 2019.** The stages of clinical disease were categorized as: (A) early stage, scratching evident and/or individual persistently scratching within a group of animals in advanced or severe stages; (B) advanced stage, difficulty walking and/or visible injuries to the limbs; and (C) severe stage, alopecia and crusting extending to several parts of the body. Because the categories represent increasing severity, each level includes the signs of the previous one.

|  | **Individuals examined** | **Individuals with mange** | **Proportion**  **(95% CI)** | **Early**  **stage** | **Advanced**  **stage** | **Severe**  **stage** |
| --- | --- | --- | --- | --- | --- | --- |
| *Age class* |  |  |  |  |  |  |
| Cria | 86 | 13 | 15.1% (8.3 – 24.5) | 10 (83%) | 2 (17%) | 0 |
| Juvenile | 137 | 37 | 27% (19.8 – 35.3) | 2 (5%) | 22 (59%) | 13 (35%) |
| Adult | 205 | 59 | 28.8% (22.7 – 35.5) | 6 (11%) | 35 (65%) | 13 (24%) |
| Not determined | 50 | 6 | 12% (4.5 – 24.3) | 1 (8%) | 9 (75%) | 2 (17%) |
| *Month* |  |  |  |  |  |  |
| February 2017 | 189 | 42 | 22.2% (16.5 – 28.8) | 7 (17%) | 19 (45%) | 16 (38%) |
| May 2017 | 70 | 25 | 35.7% (24.6 – 48.1) | 7 (28%) | 16 (64%) | 2 (8%) |
| September 2017 | 53 | 24 | 45.3% (31.6 – 59.6) | 1 (4%) | 18 (75%) | 5 (21%) |
| December 2017 | 35 | 8 | 22.9% (10.4 – 40.1) | 2 (25%) | 4 (50%) | 2 (25%) |
| April 2018 | 54 | 6 | 11.1% (4.2 – 22.6) | 0 | 4 (67%) | 2 (33%) |
| June 2018 | 43 | 8 | 18.6% (8.4 – 33.4) | 2 (25%) | 5 (63%) | 1 (13%) |
| September 2018 | 34 | 2 | 5.9% (0.7 – 19.7) | 0 | 2 (100%) | 0 |
| April 2019 | 0 |  |  |  |  |  |
| *Transect* |  |  |  |  |  |  |
| Llano de los Leones | 159 | 54 | 34% (26.7 – 41.9) | 10 (19%) | 28 (53%) | 15 (28%) |
| Agüita del Indio | 184 | 28 | 15.2% (10.4 – 21.2) | 1 (3%) | 21 (72%) | 7 (24%) |
| Caserones | 15 | 4 | 26.7% (7.8 – 55.1) | 0 | 4 (100%) | 0 |
| Llano San Guillermo | 120 | 29 | 24.2% (16.8 – 32.8) | 8 (28%) | 15 (52%) | 6 (21%) |
| **Total** | 478 | 115 | 24.1% (20.3 – 28.2) | 19 (17%) | 68 (59%) | 28 (24%) |
