# Supplementary material for: Sarcoptic mange outbreak decimates South American wild camelid populations in San Guillermo National Park, Argentina
Source: PLoS One. 2022 Jan 21;17(1):e0256616. doi: 10.1371/journal.pone.0256616 (PMC8782313; doi:10.1371/journal.pone.0256616)
Supplement: S3 Table — Interpretation example: “living vicuñas observed in December 2017 were 5.33 times more likely to present mange than living vicuñas observed in April 2018.” Asterisks indicate significant differences among levels (P < 0.05). (DOCX) [file pone.0256616.s005.docx]

**Table S3**: Odds ratio of different variable categories with regards to the occurrence of mange in living vicuñas. Interpretation example: “living vicuñas observed in December 2017 were 5.33 times more likely to present mange than living vicuñas observed in April 2018.” Asterisks indicate significant differences among levels (P < 0.05).

| **Category** | **Reference category** | **Odds Ratio** | **95% CI** |
| --- | --- | --- | --- |
| Dec 17 | Apr 18 | 5.33 | (1.39 – 20.42)* |
| Feb 17 | Apr 18 | 2.80 | (0.97 – 8.07) |
| Jun 18 | Apr 18 | 3.64 | (0.99 – 13.34) |
| May 17 | Apr 18 | 2.99 | (0.97 – 9.25) |
| Sep 17 | Apr 18 | 11.26 | (3.39 – 37.43)* |
| Sep 18 | Apr 18 | 1.22 | (0.21 – 7.18) |
| Feb 17 | Dec 17 | 0.53 | (0.20 – 1.38) |
| Jun 18 | Dec 17 | 0.68 | (0.19 – 2.51) |
| May 17 | Dec 17 | 0.56 | (0.18 – 1.72) |
| Sep 17 | Dec 17 | 2.11 | (0.74 – 6.01) |
| Sep 18 | Dec 17 | 0.23 | (0.04 – 1.32) |
| Jun 18 | Feb 17 | 1.30 | (0.46 – 3.7) |
| May 17 | Feb 17 | 1.07 | (0.52 – 2.18) |
| Sep 17 | Feb 17 | 4.02 | (1.88 – 8.62)* |
| Sep 18 | Feb 17 | 0.44 | (0.09 – 2.09) |
| May 17 | Jun 18 | 0.82 | (0.27 – 2.50) |
| Sep 17 | Jun 18 | 3.09 | (0.99 – 9.70) |
| Sep 18 | Jun 18 | 0.34 | (0.06 – 1.87) |
| Sep 17 | May 17 | 3.77 | (1.44 – 9.87) |
| Sep 18 | May 17 | 0.41 | (0.08 – 2.05) |
| Sep 18 | Sep 17 | 0.11 | (0.02 – 0.56)* |
| Caserones | Agüita del Indio | 5.77 | (1.42 – 23.33)* |
| Llano de los Leones | Agüita del Indio | 4.59 | (2.34 – 9.00)* |
| Llano San Guillermo | Agüita del Indio | 3.05 | (1.49 – 6.25)* |
| Llano de los Leones | Caserones | 0.80 | (0.21 – 3.07) |
| Llano San Guillermo | Caserones | 0.53 | (0.13 – 2.10) |
| Llano San Guillermo | Llano de los Leones | 0.66 | (0.34 – 1.31) |
| Cria | Adult | 0.51 | (0.25 – 1.01) |
| Juvenile | Adult | 0.83 | (0.46 – 1.50) |
| Juvenile | Cria | 1.65 | (0.75 – 3.62) |
