# Supplementary material for: Sarcoptic mange outbreak decimates South American wild camelid populations in San Guillermo National Park, Argentina
Source: PLoS One. 2022 Jan 21;17(1):e0256616. doi: 10.1371/journal.pone.0256616 (PMC8782313; doi:10.1371/journal.pone.0256616)
Supplement: S4 Table — Interpretation example: “living vicuñas of the “cria” age group were 4.68 times more likely to present early stage mange than living vicuñas of the “adult” age group.” Asterisks indicate significant differences among levels (P < 0.05). (DOCX) [file pone.0256616.s006.docx]

**Table S4**: Odds ratio of different variable categories with regards to the occurrence of different clinical stages of mange in living vicuñas. Interpretation example: “living vicuñas of the “cria” age group were 4.68 times more likely to present early stage mange than living vicuñas of the “adult” age group.” Asterisks indicate significant differences among levels (P < 0.05).

| **Category** | **Reference category** | **Odds Ratio** | **95% CI** |
| --- | --- | --- | --- |
| **Early stage disease** | | | |
| Cria | Adult | 4.68 | (1.48 – 14.76)* |
| Juvenile | Adult | 0.55 | (0.09 – 3.22) |
| Caserones | Agüita del Indio | Not applicable |  |
| Llano de los Leones | Agüita del Indio | 13.18 | (1.35 – 128.21)* |
| Llano San Guillermo | Agüita del Indio | 15.72 | (1.68 – 147.58)* |
| May-17 | Feb 2017 | 1.36 | (0.38 – 4.83) |
| Sep-17 | Feb 2017 | 2.61 | (0.25 – 27.39) |
| Dec-17 | Feb 2017 | 4.78 | (0.69 – 33.14) |
| Apr-18 | Feb 2017 | Not applicable |  |
| Jun-18 | Feb 2017 | 0.89 | (0.13 – 5.98) |
| Sep-18 | Feb 2017 | Not applicable |  |
| **Advanced stage disease** | | | |
| Cria | Adult | 0.14 | (0.03 – 0.62)* |
| Juvenile | Adult | 1.46 | (0.66 – 3.22) |
| Caserones | Agüita del Indio | 13.28 | (2.58 – 68.39)* |
| Llano de los Leones | Agüita del Indio | 4.6 | (1.85 – 11.45)* |
| Llano San Guillermo | Agüita del Indio | 1.27 | (0.47 – 3.45) |
| May-17 | Feb 2017 | 3.16 | (1.16 – 8.59)* |
| Sep-17 | Feb 2017 | 11.02 | (4.10 – 29.62)* |
| Dec-17 | Feb 2017 | 3.08 | (0.84 – 11.24) |
| Apr-18 | Feb 2017 | 0.91 | (0.20 – 4.16) |
| Jun-18 | Feb 2017 | 7.85 | (1.77 – 34.73)* |
| Sep-18 | Feb 2017 | 2.89 | (0.50 – 16.54) |
| **Severe stage disease** | | | |
| Cria | Adult | Not applicable |  |
| Juvenile | Adult | 1.12 | (0.43 – 2.91) |
| Caserones | Agüita del Indio | Not applicable |  |
| Llano de los Leones | Agüita del Indio | 5.68 | (1.88 – 17.13)* |
| Llano San Guillermo | Agüita del Indio | 2.39 | (0.64 – 9.00) |
| May-17 | Feb 2017 | 0.32 | (0.06 – 1.64) |
| Sep-17 | Feb 2017 | 2.25 | (0.61 – 8.21) |
| Dec-17 | Feb 2017 | 1.32 | (0.26 – 6.71) |
| Apr-18 | Feb 2017 | 0.81 | (0.15 – 4.50) |
| Jun-18 | Feb 2017 | 0.84 | (0.08 – 9.02) |
| Sep-18 | Feb 2017 | Not applicable |  |
