# Supplementary material for: Sarcoptic mange outbreak decimates South American wild camelid populations in San Guillermo National Park, Argentina
Source: PLoS One. 2022 Jan 21;17(1):e0256616. doi: 10.1371/journal.pone.0256616 (PMC8782313; doi:10.1371/journal.pone.0256616)
Supplement: S5 Table — (DOCX) [file pone.0256616.s007.docx]

**Table S5: Chi-square test summary comparing observed and expected heterozygosis for the Hardy-Weinberg equilibrium in mites from guanacos and vicuñas.**

| **Host** | **Locus** | **Degrees of freedom** | **χ^2^** | ***P-value*** |
| --- | --- | --- | --- | --- |
| Guanaco | SARM-33 | Monomorphic |  |  |
| Guanaco | SARM-34 | Monomorphic |  |  |
| Guanaco | SARM-35 | 1 | 0.543 | 0.461 |
| Guanaco | SARM-36 | Monomorphic |  |  |
| Guanaco | SARM-37 | Monomorphic |  |  |
| Guanaco | SARM-38 | Monomorphic |  |  |
| Guanaco | SARM-40 | Monomorphic |  |  |
| Guanaco | SARM-41 | Monomorphic |  |  |
| Guanaco | SARM-45 | Monomorphic |  |  |
| Guanaco | SARM-44 | 1 | 0.110 | 0.740 |
| Vicuña | SARM-33 | 3 | 12.027 | 0.007 |
| Vicuña | SARM-45 | Monomorphic |  |  |
| Vicuña | SARM-35 | Monomorphic |  |  |
| Vicuña | SARM-38 | Monomorphic |  |  |
| Vicuña | SARM-34 | Monomorphic |  |  |
| Vicuña | SARM-44 | Monomorphic |  |  |
| Vicuña | SARM-40 | 1 | 13.000 | <0.001 |
| Vicuña | SARM-41 | Monomorphic |  |  |
| Vicuña | SARM-36 | Monomorphic |  |  |
| Vicuña | SARM-37 | 1 | 0.090 | 0.764 |
